# Politics, geopolitics, and the history of science: on James Secord’s “Inventing the scientific revolution”*

**DOI:** 10.1590/S0104-59702025000100021

**Published:** 2025-05-19

**Authors:** Ana Barahona, Brian Becerra-Bressant, Diana Galván-Escobar, Lucía Granados-Riveros, Marco Ornelas-Cruces, Kapil Raj, Daniel Serrano-Juárez, Erica Torrens, Teresa Villegas-López, María Alicia Villela-González

**Affiliations:** i Professor, Facultad de Ciencias/universidad Nacional Autónoma de México. Mexico City – Mexico ana.barahona@ciencias.unam.mx; ii PhD student, Posgrado en Ciencias Biológicas/Universidad Nacional Autónoma de México. Mexico City – Mexico becerra_bressant@ciencias.unam.mx; iii Master’s student, Posgrado en Filosofía de la Ciencia/Universidad Nacional Autónoma de México. Mexico City – Mexico dianagalesco96@gmail.com; iv Master’s student, Posgrado en Filosofía de la Ciencia/Universidad Nacional Autónoma de México. Mexico City – Mexico luciagranadosriveros@gmail.com; v PhD student, Posgrado en Filosofía de la Ciencia/Universidad Nacional Autónoma de México. Mexico City – Mexico bio_marco@ciencias.unam.mx; vi Research Professor, École des Hautes Études en Sciences Sociales. Paris – France kapil.raj@ehess.fr; vii Researcher, Instituto de Geografía/Universidad Nacional Autónoma de México. Mexico City – Mexico josedanielserrano@filos.unam.mx; vii Professor, Facultad de Ciencias/Universidad Nacional Autónoma de México. Mexico City – Mexico torrens@ciencias.unam.mx; ix Master’s student, Posgrado en Filosofía de la Ciencia/Universidad Nacional Autónoma de México. Mexico City – Mexico tevilzep@gmail.com; x MSc, Facultad de Ciencias/Universidad Nacional Autónoma de México. Mexico City – Mexico aliciavillela@ciencias.unam.mx

**Keywords:** Scientific Revolution, James Secord (1953, History of science, Cold War era, Revolución Científica, James Secord (1953, Historia de la ciencia, Guerra Fría

## Abstract

Since the institutionalisation of the history of science as a discipline in the early 1950s, the “Scientific Revolution” has been its master narrative and central organising principle. However, this narrative has attracted critical scrutiny since the last decades of the twentieth century. For many historians, the idea of the Scientific Revolution was formulated when the history of science was confined overwhelmingly to North American and British institutions. Instead of focusing on Europe and the West, it might be more fruitful to recognise the influence of post-Second World War politics in shaping the concept, while also taking into account the demographic changes in the discipline, which today includes women and men from all over the world.

## Resumen

A partir de la institucionalización de la historia de la ciencia como disciplina en la década de 1950, la “Revolución Científica” ha sido su relato maestro y su principio organizador. Sin embargo, desde finales del siglo XX, esta narrativa ha sido objeto de un escrutinio crítico. Para muchos historiadores, la idea de la Revolución Científica se formuló cuando la historia de la ciencia se restringía a las instituciones norteamericanas y británicas. En lugar de centrarse en Europa y Occidente, sería más fructífero reconocer la influencia de la política posterior a la Segunda Guerra Mundial en la configuración del concepto, considerando también el cambio demográfico en la disciplina que hoy incluye a mujeres y hombres de todo el mundo.

Ever since the institutionalisation of the history of science as a discipline in the early 1950s, the “Scientific Revolution” has been both its master narrative and its central organising principle. It has commonly been accepted that the concept, elaborated during and immediately after Second World War, at once operated a revolution in the way the advancement of knowledge and science themselves were conceived and posited the founding moment for modern mathematically and experimentally based science as we know it – and, indeed, for modernity itself. In place of a model that represented science as progressing brick by brick, the Scientific Revolution introduced a radical caesura. Thanks in particular to the seminal contribution of the Russo-French philosopher Alexandre Koyré (1892-1964), science was now thought to advance through radical conceptual changes in our understanding of nature. Thus, in place of a linear accumulation of knowledge, “the mathematisation of nature with an emphasis on experience and experimentation” (Koyré, 1973, p.14) that characterised the new physics of the seventeenth century – Koyré’s epitome of modern science – constituted a radical transformation in our way of thinking about the universe. Reworked into a historical framework and popularised in the late 1940s and early 1950s by the Cambridge historians Herbert Butterfield (1900-1979) and Alfred Rupert Hall (1920-2009), the Scientific Revolution came to form the basis of the teaching of the history of science as of the 1950s, when it emerged in Anglo-American universities as a discipline in its own right, with the active support of the US State apparatus (of which we have more to say below).

However, the Scientific Revolution has inspired misgivings almost ever since its invention, attracting critical scrutiny since the latter decades of the twentieth century, as we shall see below. Exposed furthermore for its conscious adoption in order to bolster a West-centred conception of scientific superiority during the Cold War, the notion has now been rebutted on several fronts. Nevertheless, the Scientific Revolution continues to be venerated as the “big bang” moment of modern science, spawning a plethora of publications, mainly by an older generation of Anglo-American historians of science, as well as by science popularisers and Euro-supremacists (F. Cohen, 1994, 2011; Jacob, 2010; Teich, 2015; Wootton, 2015; Ferguson, 2011; Harari, 2014; see also Netz, 2022). Ever since Needham famously asked why the Scientific Revolution had taken place in the West rather than in China (Needham, 1969), this revolution (or the lack thereof) has been the central tenet used to frame and undergird the historiography of science in Asia, especially in China and India (Kim, 2004; Kak, 26 Dec. 2015). Ironically, the Scientific Revolution has recently also been a focus of many endeavours by Hispanophone historians of science to include early modern Spanish science in the grand narrative of the rise of modern science on both sides of the Atlantic (Barrera-Osorio, 2006; Cañizares-Esguerra, 2006, 2017; Olarte, 2013). As two eminent historians of science put it in a recent article, “it could be said that [Spanish-speaking historians] were boarding the flagship of modern science [i.e., the Scientific Revolution] right before it sank” (Pimentel, Pardo-Tomás, 2017, p.133). And strangely, some historians of science, although fully aware of the untenability of the concept, still feel the need to clutch onto it, much like a security blanket, in the name of a so-called “Great Tradition” (Cook, 2017). Decidedly, in the words of the historian of science Mario Biagioli, even though the Scientific Revolution has become a ghost, “institutional practices … seem to keep [it] going, if only as an ‘undead’ category” (Biagioli, 1998, p.144).

In a bizarre twist to this surreal saga, James Secord, an eminent historian of nineteenth-century science, has opened yet another front. In a recent article in *Isis* entitled “Inventing the Scientific Revolution,” he challenges the very story of the establishment of the concept by Alexandre Koyré and Herbert Butterfield in the mid-twentieth century (Secord, 2023). According to him, the term had already emerged in a different historical and intellectual context several decades earlier, namely, on the eve of First World War in the United States, where it had been popularised during the interwar period as the basis for a progressive social philosophy, principally by the preacher–historian James Harvey Robinson (1863-1936). Secord is careful to frame his argument not as a simple precursor story, but rather as a case of “brand identity,” borrowing the term from Harold Cook (2017, p.395). Conceding that the phrase “scientific revolution” (which he consciously chooses not to capitalise throughout the article [Secord, 2023, p.51, fn.1] – a point we shall come to later) had already been used since the nineteenth century to refer to the momentous changes in scientific reasoning during the sixteenth and seventeenth centuries, he aims, rather, to show that it gained wide currency only in the context of what, in United States historiography, is known as the Progressive Era, to become the founding moment of present-day modernity (Secord, 2023, p.52). In other words, Secord seeks to show that the scientific revolution had a well-established life decades before the institutionalisation of the discipline in the post-Second World War period, and it is to this earlier period that the brand name refers. Rather than “inventing the scientific revolution” *per se*, as the title suggests, the article is really about the invention of a brand name.

For readers unfamiliar with United States historiography, the interwar years saw the emergence of an attempt to use history to understand contemporary problems. Drawing heavily on John Dewey (1859-1952), the New History, as it came to be called, was characterised by efforts to further contemporary notions of social justice, women’s rights, international cooperation, world peace, and, more generally, a world freed from religion. Secord traces the first deployment of the term “scientific revolution” to Martha Ornstein (1878-1915), who used it in her 1913 thesis, *The role of scientific societies in the seventeenth century*. Subsequently borrowed by Robinson, her doctoral supervisor, the term came to serve, in Secord’s reading, as the central concept of his manifesto for the New History, *The mind in the making*, first published in 1921. This work was structured around Ornstein’s thesis that the scientific revolution had provided a critical mode of reasoning that was universally applicable, undergirding the modern age. As such, it constituted the turning point in the emergence of the modern mind.

According to Secord (2023, p.61), Robinson’s book was “one of the publishing triumphs of the century,” becoming hugely popular in the United States during the interwar years. It was, he claims, read by “hundreds of thousands” (p.66) of people and, crucially, influenced a number of textbooks adopted by many universities and schools in the country written by sociologists and historians such as Harry Elmer Barnes, James Bossard, Preserved Smith, and, in particular, the educational reformer Harold Ordway Rugg. Through these endeavours, the scientific revolution became a “significant element in progressive curricular development” (p.62).

Thus, claims Secord, when in 1943 Alexandre Koyré, who had taken refuge in New York from Nazi-occupied Paris, published his first article on the scientific revolution in English, in the *Philosophical Review*, “he was not introducing the notion, but intervening in a discussion that he knew would be familiar to [its] readers” (Secord, 2023, p.66). And when Butterfield, a British conservative and “staunch Methodist,” later deployed the term in his *Origins of modern science* (1949), he was not borrowing it from Koyré, as is generally believed, but was, in fact, referring dismissively to Robinson (Secord, 2023, p.52, 71). In effect, Butterfield’s use of “so-called” to qualify the term “scientific revolution” at its first mention in the book leads Secord to affirm that this was a disparaging reference to the work of his ideological nemesis, the progressive and liberal James Harvey Robinson. For, in Secord’s reading, Butterfield did not recognise the empirical and caesural nature of the scientific revolution, stressing instead the abstract, conceptual, and long-term processual qualities of the changes involved and thereby rendering it inapt as the bedrock for rationally inspired social change.^1^

Secord goes on to assert that it was the ensuing debates “about the scientific revolution [that] provided a platform for staging the history of science as an academic discipline” (Secord, 2023, p.72) in the post-Second World War years. However, he laments the preference, in the emerging domain, for authors such as Koyré, Burtt, Butterfield, and Whitehead, who allegedly “agonized about modern science,” over Robinson and his “progressive historian” friends, who argued for a formative and positive conception of the scientific revolution (p.73). Finally, he contends that these attempts to downplay the scientific revolution and its place in the formation of the modern mind simply resulted in a “debilitating divide between academic and public perceptions of what the history of science is about” (p.76).

However, for Secord, like it or not, the concept of scientific revolution is today almost impossible to dislodge, because while the New Historians may be consigned to oblivion, their work during the interwar years deeply inculcated the common citizen with a “scientific habit of mind” (Secord, 2023, p.76). Moreover, in recent years, the scientific revolution has been embedded even more deeply in the public mind thanks to science idolators such as Yuval Harari and Euro-supremacists of the likes of Niall Ferguson. So widespread and deeply entrenched is this belief, Secord claims, that any attempt to dislodge it would be futile. In other words, any attempt to call into question the historical validity of the scientific revolution or “to employ such potent phrases as … ‘early modern’ as [a] neutral chronological marker” would likely fail (Secord, 2023, p.76).

Secord’s article can also be read as an endeavour to claim the invention of the scientific revolution for the United States over its widely accepted appropriation by European thinkers. From this perspective, it can be seen as a nostalgic attempt to revive the sense of a lost golden age in US history, when the scientific revolution served as the backbone for a progressive social and political agenda. To this end, it also seeks to rehabilitate Robinson and his New Historian colleagues and their views, which were corrupted after Second World War: “all bundled up, forgot by all,” as Secord (2023, p.76) wistfully concludes, citing a poem by Martha Ornstein.

Overall, it must be recognised that Secord’s article is an important scholarly contribution, providing an enlightening glimpse – especially for those unfamiliar with the history of thought in the United States – of the network of intellectuals articulated not only by people such as Robinson, but also by Thorstein Veblen, Charles Beard, Carl Becker, Preserved Smith, and Harold Rugg: men who all shared and spread progressivism during the interwar years.^2^ This overview, which takes up a substantial part of the article, is particularly instructive in bringing to light the circulation of ideas in this circle of scholars and the influence of these ideas in determining and providing material for progressive school curricula in social studies and history in the United States during the interwar years.

Given the above, it is rather puzzling to observe that Secord, while detailing Harold Rugg’s seminal role in this formidable project and largely portraying him as its central figure, seems to attribute all the credit to a single person: Robinson (Secord, 2023, p.63-75, passim). Furthermore, his description fails to demonstrate that the idea of the scientific revolution gained widespread currency during this period and, if it did, whether this was attributable only to Robinson and his close circle. For, in focusing exclusively on these actors, Secord does not mention the contributions of other authors who used the term. These include Robinson’s own inspiration, John Dewey, who, in his *German philosophy and politics* (1915), preceded Robinson in his usage of the term by at least six years; not to mention Alfred North Whitehead, in *Science and the modern world* (1925); or even *Making of the modern mind* (1926), by the historian of philosophy John Herman Randall Jr., to pick a few well-known examples. Also, it is important to point out that the idea of the scientific revolution ran a different, parallel course in Britain, where it was more the preserve of Marxist intellectuals, notably John Desmond Bernal and Harold Laski.

Evidently, one could argue, as Secord seems to do, that Robinson and his friends were more successful than the others in introducing the idea to the popular repertoire, in that they were instrumental in introducing a progressive curriculum, with accompanying textbooks, to US junior high schools. However, Secord fails to demonstrate that their ideas – particularly the notion of the scientific revolution – did actually gain wide currency in the United States in the interwar years. He provides no evidence of references to the scientific revolution in lay or scholarly publications from this period, nor any reference to the reception of the new historical corpus. By the same token, we have no choice but to take his word that the scientific revolution had a definite influence on future historians of science. His assertion that it was “almost certainly” in the progressive school Thomas Kuhn attended that, as a 15-year-old, he “first learned about the scientific revolution” (Secord, 2023, p.66), is scarcely credible. He provides no evidence for this, except a single reference to a reminiscence by Kuhn at the end of his life that his school had been “‘particularly good in terms of teaching me to think for myself’” (p.66). Despite being a prolific writer on the path to his caesural conception of science, Kuhn himself never once mentions Robinson or any of his entourage in any of his writings, instead repeatedly acknowledging his intellectual debt to US and European thinkers; in particular, Alexandre Koyré.

I. Bernard Cohen (1914-2003) – the first professor of the history of science at Harvard and one of the pioneers of the discipline, who also grew up during the interwar years – also explicitly acknowledged Koyré’s seminal role in introducing the Scientific Revolution (note the capitalisation here) to the United States (I.B. Cohen, 1987; see also Pisano, Agassi, Drozdova, 2018). Even though he is known to have been familiar with Robinson’s use of the notion, which he mentions in his authoritative *Revolution in science*, Cohen writes that “prior to about 1950, despite the occurrence of easily locatable individual statements about both the Scientific Revolution and particular revolutions in science, neither of these ideas was used in a significant way to organize the historical discussion. Nor was there much interest shown in the question of whether there are revolutions in science, or in the nature and structure of such revolutions if they actually occur” (I.B. Cohen, 1985, p.402). The watershed moment, for Cohen, was “to a large degree an effect of the influence of Butterfield and Koyré” (p.402-403). Despite being widely used earlier, the term “scientific revolution” was for Cohen a placeholder, a concept devoid of any specific content.

How, then, can Secord justify his contention that this “intellectual bomb,” as he calls the introduction of the scientific revolution, came earlier, thanks to Robinson and the New Historians? We believe this is possible because his criterion for making his claim is very different from that of Cohen’s. For, whereas Cohen’s yardstick for measuring the spread of the idea is the role it played in structuring the academic milieu of historians of science, Secord’s is its supposed popularity amongst the broader US population, making it the founding moment of a rational age that marks the triumph of science over religion. There are two important observations to be made with respect to this here.

Firstly, lacking any specific power to organise and render the phenomenon of modern science meaningful, as in Cohen, Secord’s rendering of the scientific revolution is thus no more than a chronological marker for Robinson and the New Historians: an alternative to the Renaissance–Reformation–Restoration trio (all strongly associated with Europe and Christianity) with a non-religious overtone more in tune with their own ideology. It is quite possibly because the concept was indeed nothing other than a placeholder, as Cohen believed, that the New Historians chose not to capitalise the “scientific revolution.” In not seeing this important distinction and choosing to follow the New Historians’ convention throughout his text, Secord thereby confounds these two contrasting conceptions.

Secondly, as we remark above, Secord provides no evidence to support his claim of the supposed popularity of the idea of the scientific revolution. We thus cannot measure its real impact among the interwar generations in the United States, let alone elsewhere in the world. The number of reprints, editions, and translations of Robinson’s book, while providing some indicator of its general popularity, tells us little about the specific acceptance of the “scientific revolution.” In order to gauge its popularity, one would need at least some idea of references to Robinson in relation to the term by other authors or in journals or other publications.

Notwithstanding, Secord ventures further, projecting the New Historians’ “scientific revolution” as a solidly anchored brand identity. He thus imagines that the scientific revolution has continued to be significant in the popular imagination ever since the 1930s. In other words, he makes two unsubstantiated assumptions: that the New Historians’ conception of the scientific revolution was widely popular, and that it remains so.

It is our contention, however, that even if the term was widely accepted in the interwar years, its meaning was radically transformed in the wake of Koyré and Butterfield, and that this change was not simply due to intellectual debates between two scholarly groups, as Secord (2023, p.71) would have it. Rather, it was the result of an overt attempt spearheaded by the United States to create a scholarly discipline that could serve as a powerful ideological weapon in the years immediately following Second World War, which was also expressed through the progressive capitalisation of “Scientific Revolution.”

In order to elaborate on our point, let us move away from Secord’s focus on Butterfield’s supposedly dismissive “so-called” to qualify the scientific revolution (still used in the lower case by Butterfield) and take a closer look at his *Origins of modern science* and the context of its publication in 1949; that is, in the wake of Britain’s Pyrrhic victory in Second World War and the dawn of a post-imperial future with an ever-diminishing role for the Old Continent on the global scene. A prominent anti-Whig, anti-Marxist Cambridge historian, Butterfield was seeking to assert western Europe’s cultural and intellectual superiority. Reworking Koyré’s rupturist epistemological concept into a historical framework, he thus declared that “it [the scientific revolution] outshines everything since the rise of Christianity and reduces the Renaissance and Reformation to the rank of mere episodes, mere internal displacements within the system of medieval Christendom” (Butterfield, 1949, p.viii). Putting to rest any doubt there may have been about the geographical location of this sea change, he devoted a whole chapter to its place in history, writing:

The scientific revolution we must regard, therefore, as a creative product of the West – depending on a complicated set of conditions which existed only in western Europe … And when we speak of Western civilisation being carried to an oriental country like Japan in recent generations, we do not mean Græco-Roman philosophy and humanist ideals, we do not mean the Christianising of Japan, we mean the science, the modes of thought and all that apparatus of civilisation which were beginning to change the face of the West in the latter half of the seventeenth century (Butterfield, 1949, p.163).

On the other side of the Atlantic, there was a different story with even stronger geopolitical overtones. It is a story worth detailing here, despite having been sketched out in previous publications by one of the co-authors of this article (Raj, 2017, 2024). The history of science was indeed created by *fiat* at the highest levels of the US State apparatus in the second half of the 1940s, with the Scientific Revolution (capitalised) as its cornerstone. It was weaponised in the Cold War world of ruthless competition between the West and the Soviet bloc and was instituted as part of a concerted effort under the aegis of the US Office of Scientific Research and Development. Collaborating in this effort were James B. Conant (1893-1978), who was a chemist, a onetime chairman of the Manhattan Project, president of Harvard University from 1933 to 1953, the first US ambassador to West Germany, and one of the principal architects of the Marshall Plan and NATO, and Karl T. Compton (1887-1954), a physicist, president of MIT from 1930 to 1948, president of MIT Corporation from 1948 until his death, scientific and military advisor to President Roosevelt, and unrepentant defender of the bombing of Hiroshima and Nagasaki. With the active participation of I. Bernard Cohen and Henry Guerlac (1910-1985) – two of Koyré’s transatlantic admirers, who founded the discipline in the United States – the history of science was consciously seized upon to construct a Koyré/Butterfield-inspired narrative according to which the (capitalised) Scientific Revolution had set Western civilisation on a unique course of free and critical thinking; a revolution that supposedly undergirded modern Western democracies and was the rightful heir to the post-Galilean values of enlightened liberalism that fundamentally distinguished them from all other cultures and civilisations, particularly the Soviet Union, but also the emerging postcolonial world (Backhouse, Maas, 2017).

Along with NATO, the Marshall Plan, and the Bretton Woods Conference, all of which were designed to rein in Europe as part of a coherent Western alliance, another set of institutions and policies was put in place to attract and domesticate the rest of a progressively expanding post-colonial world. These included the World Bank and the United States Agency for International Development, to name but two. The US higher education system was also requisitioned to do its bit in spreading the ideals of Western civilisation, receiving lavish funding and new teaching and research positions. It was this West-supremacist credo that was at the heart of the history of science in its introduction and institutionalisation as an independent field of learning in the early 1950s in both US and British universities. The potential of Butterfield’s conception to neatly encapsulate the uniquely scientific basis and superiority of Western civilisation in relation to all others, especially as a counter to the communism of the Soviet Union, helped put it at the centre of its teaching. The history of science, fashioned around the Scientific Revolution in order to highlight the pioneering role of the West, was to form an integral part of this panoply to ensure US-backed Western hegemony over what came to be called the “Third World” and keep it away from Soviet influence (Hollinger, 1995).

However, if Koyré’s and Butterfield’s writings had provided the intellectual backbone of the fledgling discipline, the iconic text for the generations of history of science students that followed in Britain and the United States was that of another British historian of science, Alfred Rupert Hall, who had discovered Koyré independently in the course of his early research work in the United States in the late 1940s.^3^ Indeed, Hall’s *Scientific Revolution, 1500-1800*, published in 1954, was the text around which post-Second World War history of science first coalesced (Hall, 1954)*.* While giving historical flesh and bones to Koyré’s idealistic construct of the scientific revolution, this book was even more decisively Euro-supremacist than Butterfield’s. At the very outset, it explicitly expresses European superiority over the rest of the world: “European civilization at the beginning of the sixteenth century was isolated,” but “during the next centuries the trend of the middle ages [*sic*] was to be reversed; Europeans were to teach the East far more than they learned from it” (Hall, 1954, p.1-2).

Hall’s book was soon complemented by contributions from the first generation of US historians of science. These contributions fleshed out the discipline’s construct of this Western-supremacist notion of modern science, based on the Scientific Revolution, in a way that was very much in tune with the hubristic post-war atmosphere in the United States, which had given rise to works like William McNeill’s *Rise of the West* (1963). Below are some choice extracts from the writings of such eminent members of the community as Charles Gillispie (Secord’s doctoral supervisor), Thomas Kuhn, and Richard Westfall:

The hard trial will begin when the instruments of power created by the West come fully into the hands of men not of the West, formed in cultures and religions which leave them quite devoid of the western sense of some ultimate responsibility to man in history. That secular legacy of Christianity still restrains our world in some slight measure, however self-righteous it may have become on the one side, and however vestigial on the other. Men of other traditions can and do appropriate our science and technology, but not our history and values. And what will the day hold when China wields the bomb? And Egypt? Will Aurora light a rosy-fingered dawn out of the East? Or will Nemesis? (Gillispie, 1960, p.8-9).^4^

Every civilization of which we have records has possessed a technology, an art, a religion, a political system, laws, and so on. In many cases, those facets of civilization have been as developed as our own. But only the civilizations that descend from Hellenic Greece have possessed more than the most rudimentary science. The bulk of scientific knowledge is a product of Europe in the last four centuries. No other place and time has supported the very special communities from which scientific productivity comes (Kuhn, 1962, p.167-168).

The Scientific Revolution was the most important ‘event’ in Western history, and a historical discipline that ignores it must have taken an unhappy step in the direction of antiquarianism. … nothing [in the long list of scientific and technical achievements] is thinkable without the Scientific Revolution of the sixteenth and seventeenth centuries (Westfall, 1986, p.1).

So was the Scientific Revolution popularly associated, via a resurrection of the sixteenth and seventeenth centuries, with the Anglo-US geopolitics of the Cold War, together with an inherent sense of the cultural superiority of the West – itself a cultural construct. It was invented, just as the history of science had been created as a university discipline, to claim ownership of science for the United States and Europe and to serve as the spearhead in a larger strategy, crafted primarily by the United States, to weaponise the social sciences, especially by mobilising the concepts of modernity and modernisation, also creations of the same epoch (Knöbl, 2017; also Engerman, 2010; Solovey, Dayé, 2021). Subsequently reified and reinforced by successive generations of Anglo-US historians of science, it took on a life of its own, making it well-nigh impossible for those countries’ historians of science to think without it. In the words of the eminent French historian of science Ernest Coumet (1987, p.498), “the professionalisation of the history of science has led to the production of classic subjects, and God knows ‘the scientific revolution of the sixteenth and seventeenth centuries’ has become one of them; as a result, A. Koyré’s formulae have the ‘obviousness’ of those learned at school.”^5^ Furthermore, the Scientific Revolution has come to serve as a measure to gauge when and how different branches of science and, indeed, entire societies have broken radically with their respective pasts, that is, had their Scientific Revolutions.

To sidestep this essential episode in the institutionalisation of the history of science, ordained by the highest offices of the post-Second World War US State apparatus, with the active participation of some of the most eminent historians of science of the time, and retell the story simply in terms of an intellectual debate between two opposing academic camps is a questionable manoeuvre to say the least. Having situated it in its broader context, it would be worth examining what effect the (newly capitalised) Scientific Revolution – so widely disseminated in the United States and Britain – actually had on popular perceptions of modern science, including whether or not it actually supplanted the earlier iteration (of the New Historians). Of interest is the fact that popular science idolators and Euro-supremacists of the likes of Harari and Ferguson have chosen to use the capitalised version of the term, with all its ideological baggage.

It must also be pointed out that the concept of the Scientific Revolution did not find immediate acceptance in continental Europe. As Secord himself remarks (without pondering on its significance), even as late as 1956 the noted Dutch historian of science Eduard Jan Dijksterhuis (1892-1965) was quite circumspect about it. Nor was the Dutchman alone: many of his peers in France and elsewhere on the Continent saw little use for the concept. It was only a couple of decades later that the Scientific Revolution found its place in European and world academia, as Secord (2023, p.73) also points out. However, it would be interesting to see whether it has indeed succeeded in supplanting other chronological markers, notably the Enlightenment, which still enjoy a healthy life in European historiography.

Even in the United States and Britain, as mentioned at the start of this paper, the idea of the Scientific Revolution has not been without its critics within the community of historians of science. For even as it was established as the bedrock of the narrative for the discipline, shored up through textbooks and undergraduate syllabuses used in these countries, it almost immediately became a subject of close scrutiny. One of the major critiques consisted in the nature of the alleged new science was its mathematical and experimental nature, which identified it more with physics than any other branch of learning, as Margaret Osler (2000) argued in a compelling reappraisal published almost 25 years ago. In his review of this book, the historian of medicine and science Harold Cook stated the general consensus that “17th-century science was not modern, and that not even Newton was ‘ahead of his time’,” adding that “the forces of confusion have taken over the ivory tower, leaving us no clear idea about the origins of modern science” (Cook, 2001, p.313; see also: Hunter, 1981; Porter, 1986, p.290-316; Lindberg, Westman, 1990; Dobbs, 1994; Shapin, 1996). Not long afterwards, the editors of volume 3 of the *Cambridge History of Science*, Katherine Park and Lorraine Daston, explained why they believed the term “The Scientific Revolution” was no longer appropriate to designate the period between the sixteenth and seventeenth centuries, covered in the volume:

The cumulative force of the scholarship since the 1980s has been to insert skeptical question marks after every word of this ringing three-word phrase, including the definite article. It is no longer clear that there was any coherent enterprise in the early modern period that can be identified with modern science, or that the transformations in question were as explosive and discontinuous as the analogy with political revolution implies, or that those transformations were unique in intellectual magnitude and cultural significance (Park, Daston, 2006, p.12-13).

In short, while it is recognised that the sixteenth and seventeenth centuries were marked by important changes in the contents of knowledge and ways of knowing, there is a general consensus that it is problematic to characterise these as revolutionary.

But there is another equally, if not more important reason for critiquing the Scientific Revolution from outside the history of science community. Indeed, the notion was formulated at a time when the discipline was confined overwhelmingly to North American and British institutions, themselves predominantly the preserve of white men who were, we dare say, primarily focused on the lives and achievements of their white male predecessors. However, the demographic composition of these institutions has drastically changed. Over the past decades, they have increasingly included women of different origins. Concomitantly, their institutional geography has also been greatly enlarged to encompass a number of non-Western countries from almost all the continents. What is more, many of these women and men have worked on early modern periods and have not taken kindly to the diffusionist and gender-insensitive perspective of the spread of modern science from Europe to the rest of the world in the light of European expansion, as peremptorily laid out by George Basalla at the height of the Cold War. Indeed, in 1967, in the columns of the prestigious review *Science*, Basalla presented his simple heuristic model to the journal’s readership, which, it should be noted, included (and includes) US law and policy makers, in the following terms:

A small circle of Western European nations provided the original home for modern science during the 16th and 17th centuries: Italy, France, England, Austria, and the Scandinavian countries. The relatively small geographical area covered by these nations was the scene of the Scientific Revolution which firmly established the philosophical viewpoint, experimental activity, and social institutions we now identify as modern science ... Until fairly recent times, any region outside of Western Europe received modern science through direct contact with a Western European country (Basalla, 1967, p.611).

The conceit of this Euro-supremacist model and the Euro-US claim over its ownership acted as a red rag, almost immediately attracting historically founded critical responses with strong political and moral dimensions, predominantly from the non-West. Indeed, it is in this context that one might also understand the reaction by many Hispano-American historians of science to claim the Scientific Revolution for the Iberian world (Barrera-Osorio, 2006; Cañizares-Esguerra, 2006; Olarte, 2013).^6^ However, in recent years, the Latin American historiography of science has increasingly turned its attention to transnational connections and long-range intercontinental circulations of knowledge (Silva, Cueto, 2022). In addition to providing compelling demonstrations of the racial and economic discriminations at work in science in the colonial context, these critiques have shone a light on how the diffusionist model occludes the active processes of negotiation and differential appropriation operated by receiving groups in scientific and technological circulations. More importantly – and breaking away from the stranglehold of the Scientific Revolution paradigm – they have also persuasively shown that northwestern Europe was far from being the only region to experience dramatic changes in ways and forms of knowing (Eamon, 2009). New forms of knowledge about language, the natural world (including plants, minerals, animal life, and medicine), and mathematics, applied to metrology, timekeeping, astrology, the calendar, space, mapping, State taxation, and navigation, were emerging more or less simultaneously in many other parts of the world as well, from the Americas to Asia, passing through large parts of Africa (Sarton, 1951; Mundy, 1996; Hountondji, 1997; Hostetler, 2001; Yonemoto, 2003; Raj, 2007; Küçük, 2017; Barrera-Osorio, Olarte, 2019).

After much struggle and debate, the term “early modern” has now been increasingly adopted by historians of the period to make sense of these concomitant sea changes in social, political, and cultural organisations among far-flung polities in what can only be described as a global conjuncture (Subrahmanyam, 1997). Historians of science have also begun to follow suit in adopting this terminology, thus opening the way to finding more appropriate chronological markers for science as a global phenomenon (Shapin, 1996; Park, Daston, 2006; Küçük, 2017; Raj, 2017).

It is clear that the Scientific Revolution as we know it today was definitely not what was forged in the interwar years by Robinson and his friends. Although one can admit that the New Historians played an important role in integrating the social sciences associated with notions of social justice in the school curricula of the United States, it is also important to historically situate their thoughts and their limits, notably the idea of progress then so in vogue, as well as the scientism that undergirded their *Weltanschauung*. Whatever their real success in imposing the scientific revolution may have been, we must remember that what was branded “progressive” in the interwar years was itself historically situated and perhaps does not correspond to present-day ideals. Furthermore, the larger history and politics of the idea of the Scientific Revolution far surpass the narrow bounds of the United States and Britain, even though the concept was created within the north-Atlantic space of political and intellectual circulations. In view of the massive investments made around the post-Koyré/Butterfield notion to ensure its institutionalisation and popularisation, the capitalised Scientific Revolution gained a worldwide brand identity, and it is quite probable that public perceptions in the United States of the Scientific Revolution – as well as of science itself – underwent a corresponding change. It is indeed important to note more generally, as any philologist (and historian) will know, that the meanings and connotations of words and concepts in every language have always changed in time, the “Scientific Revolution” being no exception.

The world has today moved on from the bipolar Cold War era, with its “Third World” regarded as free for the taking by both protagonists. Instead of obsessively focusing on Europe and the West and continuing to reduce the question of scientific change to a parochial wrangle between the two sides of the North Atlantic, it might be more fruitful for Anglo-US scholars and the journals that publish them to take account of both the influence of post-Second World War politics in crafting the Scientific Revolution and the global composition of scholars in the discipline. The survival of the history of science depends on our renewing our perceptions of science and its place in our world. It is high time we buried the “ghost” (Biagioli, 1998, p.144) of the scientific revolution (with and without capitals) and started to work towards more inclusive and appropriate chronological frameworks capable of rendering visible the multiple cultural interactions at work in the making of scientific knowledge now as much as in the early-modern and modern periods. This can only be achieved through a collaborative effort to seek alternative narratives that involve the international community of historians and historians of science, technology, medicine, and the humanities, as well as members of the social sciences in general.

## Data Availability

Not deposited in a data repository.

## NOTES

1 See, however, I.B. Cohen (1985, p.398), Wootton (2015, p.17), and Cook (2017, p.398).

2 We have intentionally used the masculine noun, leaving out Martha Ornstein, who did not form part of this post-First World War movement, having died in 1915. In addition, she was cited only by Robinson and finds no mention in any other work of the period.

3 According to Hall himself, it was the refreshing contrast of Koyré’s *Études galiléennes* to “the dull treatment of the history of science that [he] found in so many articles and books, essentially consisting of the establishment of facts” that had attracted him to the history of science in the first place (Hall, c.1981; see also: I.B. Cohen, 1987, p.62; Hall, 1963, p.1-16).

4 Gillispie wrote this barely 15 years after the US atomic bombings of Hiroshima and Nagasaki, an irony that will not escape the attentive reader. Furthermore, his metaphorical choices at the end of the quotation are also deeply ironical: unbeknownst to him, Nemesis and Aurora were two of the deadliest warships in modern history. Nemesis was the first iron-clad steam-powered naval ship to wreak havoc during the First Opium War (1839-1842), killing thousands of Chinese, who referred to it as the “devil ship.” A century later, HMS Aurora was a Royal Navy destroyer that served during Second World War and was later given to the Chinese as compensation for ships looted from them by the British and lost during the war.

5 Our translation. Original citation in French: “La professionnalisation de l’histoire des sciences a entrainé la fabrication de sujets classiques, et Dieu sait si ‘la Révolution scientifique des XVIe et XVIIe siècles’ en est devenu un; du coup, les formules d’A. Koyré ont ‘l’évidence’ des formules apprises scolairement.”

6 For instance, for Jorge Cañizares-Esguerra (2017), “the narrative of the Scientific Revolution is an extraordinary instrument of political-cultural domination” (p.425), which “created a strange and parochial narrative that erased entire regions and periods” and was characterised by “the movement of objects as well as the erasure of memory” (p.431). Others, such as Antonio Barrera-Osorio (2006), go further by claiming that the scientific revolution did not begin with Copernicus and his heliocentric model, but “started in the 1520s, in Spain, when merchants, artisans, and royal officials confronted new entities coming from the New World and had to devise their own methods to collect information about those lands” (p.2).
